# Evaluation of (sdLDLc*HCYc)/HDLc ratio in clinical auxiliary diagnosis of primary cerebral infarction

**DOI:** 10.1186/s12872-022-02969-z

**Published:** 2022-12-06

**Authors:** Chunhua Luo, Yucheng Luo, Qin Ma, Chunyan Chen, Sheng Xian, Feng Gong, Wu Zhao, Jingjing Zeng, Jun Luo

**Affiliations:** 1 The First College of Clinical Medical Science, China Three Gorges University, Yichang, China; 2 Yichang Central People’s Hospital, Yichang, China

**Keywords:** (sdLDLc*HCYc)/HDLc ratio, Indicator, Auxiliary diagnosis, Cerebral infarction

## Abstract

**Background:**

Timely detection of cerebral infarction is of vital importance in planning intervention effect of rapid rehabilitation. The clinical auxiliary diagnosis value of single biomarker, including small dense low-density lipoprotein concentration (sdLDLc), homocysteine concentration (HCYc) and high-density lipoprotein cholesterol concentration (HDLc) for cerebral infarction has been confirmed by many studies. Whether the use of three biomarkers in combination by calculating (sdLDLc*HCYc)/HDLc ratio could improve the diagnosis ability for primary cerebral infarction remains to be unclear. In the present study, we conducted a cross-sectional study to evaluate the value of (sdLDLc*HCYc)/HDLc ratio in clinical auxiliary diagnosis of primary cerebral infarction.

**Methods:**

A total of 583 participants, including 299 healthy participants as control group and 284 participants diagnosed with first cerebral infarction as experiment group, were included in this respective study. The serum sdLDLc, HDLc and HCYc were measured by peroxidase method, enzyme‐linked immunosorbent assay and an enzymatic method, respectively.

**Results:**

The average concentration of sdLDL and HCY (0.69 ± 0.29 mmol/L and 18.14 ± 6.62 μmol/L) in experiment group was significantly higher than those in the control group (0.55 ± 0.22 mmol/L and 10.77 ± 2.67 μmol/L, P < 0.05). However, the average concentration of HDL (1.47 ± 0.25 mmol/L) in the control group was higher than that in the experiment group (1.33 ± 0.28 mmol/L, P < 0.05). Spearman correlation coefficient showed the three indicators are independent of each other. The positive predictive value of (sdLDLc*HCYc)/HDLc ratio (61.27%, 95% CI: 55.31–66.92) is higher than that in single biomarker (sdLDLc: 6.69 95% CI: 4.19–10.42, HCYc: 38.38%, 95% CI: 32.75–44.33, HDLc: 3.87%, 95% CI: 2.05–7.02). Receiver-operating characteristic curve (ROC) analysis illustrated that predictive power of (sdLDLc*HCYc)/HDLc was higher than single biomarker, including sdLDLc, HCYc and HDLc, in primary cerebral infarction.

**Conclusions:**

Therefore, (sdLDLc*HCYc)/HDLc ratio might be a better new indicator in clinical auxiliary diagnosis of primary cerebral infarction, which could be contributed to predicting cerebral infarction occurrence and provide a scientific basis for early prevention.

## Introduction

Cerebral infarction, with extremely high rate of disability and fatality worldwide, is one of the causes of stroke in the brain [1, 2]. It was reported that about 6.17 million people died from cerebral infarction in 2017 in the world and the mortality/disability rate in China might reach 34.5–37.1% in in-patients with acute cerebral infarction [3, 4]. Notablely, it is still hard to take effective way to reduce the mortality of acute cerebral infarction. Unfortunately, many patients diagnosed as primary cerebral infarction have developed into severe type. Therefore, timely diagnosis of cerebral infarction is of vital importance in planning intervention effect of rapid rehabilitation.

Small low-density lipoprotein (sdLDL) that is associated with metabolic disease belongs to LDL-3 to 7 [5, 6]. An increased level of sdLDLc has a certain predictive value for cardiovascular and cerebrovascular events and the National Cholesterol Education Program (NCEPIII) lists sdLDL as one of the risk factors for coronary artery disease (CAD) [7]. Many studies indicated that the sdLDLc change was positively associated with the presence of cardiovascular disease (CVDs) including cerebral infarction [8, 9].

High density lipoprotein cholesterol (HDL-C) could remove cholesterol from peripheral tissues, particularly the arterial wall [10], and is regarded as protective substance. The HDL-C level in blood is associated with cerebrovascular diseases [11].

The association of LDLc to HDLc ratio with cardiovascular disease have been studied [12, 13]. Among these, LDLc/HDLc ratio was even found to be superior to either LDLc or HDLc alone.

Homocysteine (HCY) is an endogenous sulfur containing amino acid, which represents an intermediate in methionine metabolism [14, 15]. HCY is significantly correlated with CVDs, mainly including heart attacks and strokes.

As described above, the clinical auxiliary diagnosis value of single biomarker, including sdLDLc, HCYc and HDLc for cerebral infarction has been confirmed by many studies. Whether the use of three biomarkers in combination by calculating (sdLDLc*HCYc)/HDLc ratio could improve the diagnosis ability for primary cerebral infarction remains to be unclear. Therefore, in the present study, we conducted a cross-sectional study to evaluate the value of (sdLDLc*HCYc)/HDLc ratio in clinical auxiliary diagnosis of primary cerebral infarction.

## Materials and methods

### Patient information

**Fig. 1:**
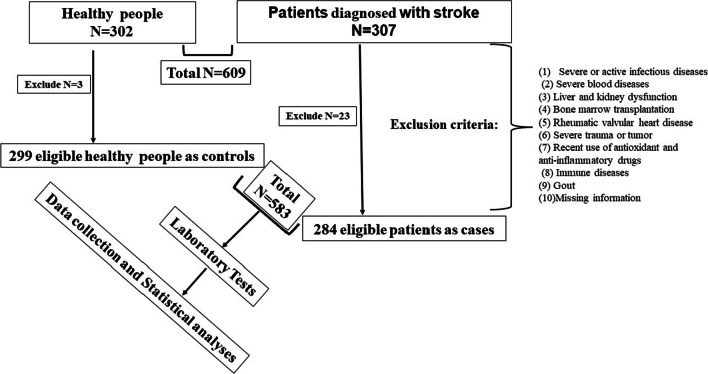
Study routine

The retrospective study aimed to investigate the value of (sdLDLc*HCYc)/HDLc ratio in clinical auxiliary diagnosis of primary cerebral infarction and was conducted at the Yichang Central People’s Hospital, China, which was approved by the Ethics Committees of Yichang Central People’s Hospital. As shown in Fig. 1, in the current study, 26 patients were excluded from this study because they were incomplete or failed to meet the quality control standards required. A total of 583 patients, including 284 patients diagnosed with primary cerebral infarction as the experimental group and 299 healthy volunteers as the control group, were included in the final analyses. The sample size calculation is based on PASS 15. The minimum sample size (alpha was set as 0.25 and power was set as 1) should reach 152. The ultimate statistical power is 1 when the sample size in this study are 284 patients in the experimental group and 299 patients in the control group (284/299 = 0.95, close to 1), which indicates the follow-up research results are feasible. Medical records were also reviewed to obtain detailed demographic and clinical information.

### Inclusion and exclusion criteria

The inclusion criteria for the patients were as follows: (1) the diagnosis of cerebral infarction was made according to the criteria from the American Stroke Association. (2) Patients present with primary cerebral infarction. (3) No history of cerebral infarction and family medical history was presented in the control group; exclusion criteria: (1) severe or active infectious diseases. (2) Severe blood diseases. (3) Liver and kidney dysfunction. (4) Bone marrow transplantation. (5) Rheumatic valvular heart disease. (6) Severe trauma or tumor. (7) Recent use of antioxidant and anti-inflammatory drugs. (8) Immune diseases. (9) Gout. (10) Missing information.

### Laboratory tests

Venous blood (3 mL) was obtained from both control group and experiment group by routine venipuncture in the early morning and centrifuged at 1500 r/min for 10 min at 4 °C. The patients underwent blood examinations in a fasting state, including routine blood examination, blood glucose, HDL, LDL, sdLDL, triglyceride (TG), total cholesterol (TC), HCY. Kits are mainly purchased from Beijing Strong Biotechnologies, Inc. The operation is based on the kit instruction description.

### Statistical analyses

Data approximately that follow a normal distribution is represented as the mean with standard deviation. Skew distribution data is represented by median and its range. Count data is represented by frequency and composition ratio. The comparison of differences between normally distributed data groups was performed by t-test or analysis of variance. Non-normal measurement data were compared based on non-parametric rank sum test. The chi-square test was used to compare the differences between groups of count data. Correlation analysis is based on spearman correlation.

Multiple linear regression analysis is used in this study. The IBM SPSS statistics 19 and GraphPad Prism 6.0 were used for analysis, and a p-value of < 0.05 was considered statistically significant.

## Results

### Baseline data

The baseline characteristics of the patients that met our eligibility criteria are shown in Table 1. Among these participants, the mean (SD) age was 64.87 ± 9.68 years and 66.90% were men in cerebral infarction group. The mean (SD) age was 47.47 ± 8.20 years and 68.89% were men in the control group. The concentration of sdLDL and HCY in cerebral infarction group were significantly higher than those in the control group (P < 0.05). The concentration of HDL in cerebral infarction group was significantly lower than this in the control group (P < 0.05). The ratio of (sdLDLc*HCYc)/HDLc in cerebral infarction group was significantly higher than those in the control group (P < 0.05). As shown in Table 1, the hemogram parameters including platelets, lymphocytes, neutrophils and monocytes are significantly different between patients in cerebral infarction group and healthy people in control group.

**Table 1:** Characteristics of patients in cerebral infarction group and healthy volunteers in control group

	Cerebral infarction n = 284 Disease	Control n = 299 Control	P value cerebral infarction versus control
Male (%)	190 (66.90)	206 (68.89)	< .0001
Age	64.87 ± 9.68	47.47 ± 8.20	< .0001
CHOL (mmol/L)	4.81 ± 1.51	4.79 ± 0.59	< .0001
TG (mmol/L)	1.53 ± 0.72	1.27 ± 0.36	< .0001
HDL (mmol/L)	1.33 ± 0.28	1.47 ± 0.25	< .0001
LD L(mmol/L)	2.46 ± 0.68	2.40 ± 0.32	0.0003
sdLD L(mmol/L)	0.69 ± 0.29	0.55 ± 0.22	0.01
GLU (mmol/L)	5.79 ± 1.63	4.70 ± 0.39	< .0001
UA (mmol/L)	339.13 ± 81.70	329.97 ± 59.07	0.01
hs CRP (mg/L)	7.54 ± 8.08	1.82 ± 0.93	< .0001
HCY(μmol/L)	18.14 ± 6.62	10.77 ± 2.67	< .0001
HbA1C(mmol/mol)	6.26 ± 1.18	5.31 ± 0.31	< .0001
LP-pla2(ng/ml)	606.20 ± 156.44	365.82 ± 145.04	0.001
sdLDLc/HDLc	0.56 ± 0.26	0.37 ± 0.15	< .0001
(sdLDLc*HCYc)/HDLc	10.26 ± 5.90	4.65 ± 1.82	< .0001
Platelet(10^9^/L)	189.40 ± 47.46	211.99 ± 43.00	< .0001
Neutrophil(10^9^/L)	4.53 ± 1.62	3.27 ± 0.72	< .0001
Lymphocyte(10^9^/L)	1.60 ± 0.44	1.94 ± 0.44	< .0001
Monocyte(10^9^/L)	0.52 ± 0.14	0.41 ± 0.09	< .0001

Categorical variables were expressed as numbers and proportions (%), continuous variables as mean ± standard deviation

*CHOL* total cholesterol; *TG* triglycerides; *HDL* high-density lipoprotein; *LDL* low-density lipoprotein; *sdLDL* small dense low-density lipoprotein; *GLU* glucose; *UA* uric acid; *hsCRP* hypersensitive-c-reactive-protein; *HCY* homocysteine; *HbA1C* Glycosylated Hemoglobin; *LP-PLA2* lipoprotein-associated phospholipase A2

### Correlation analysis of three biomarker pairs in control group and cerebral infarction group

**Fig. 2:**
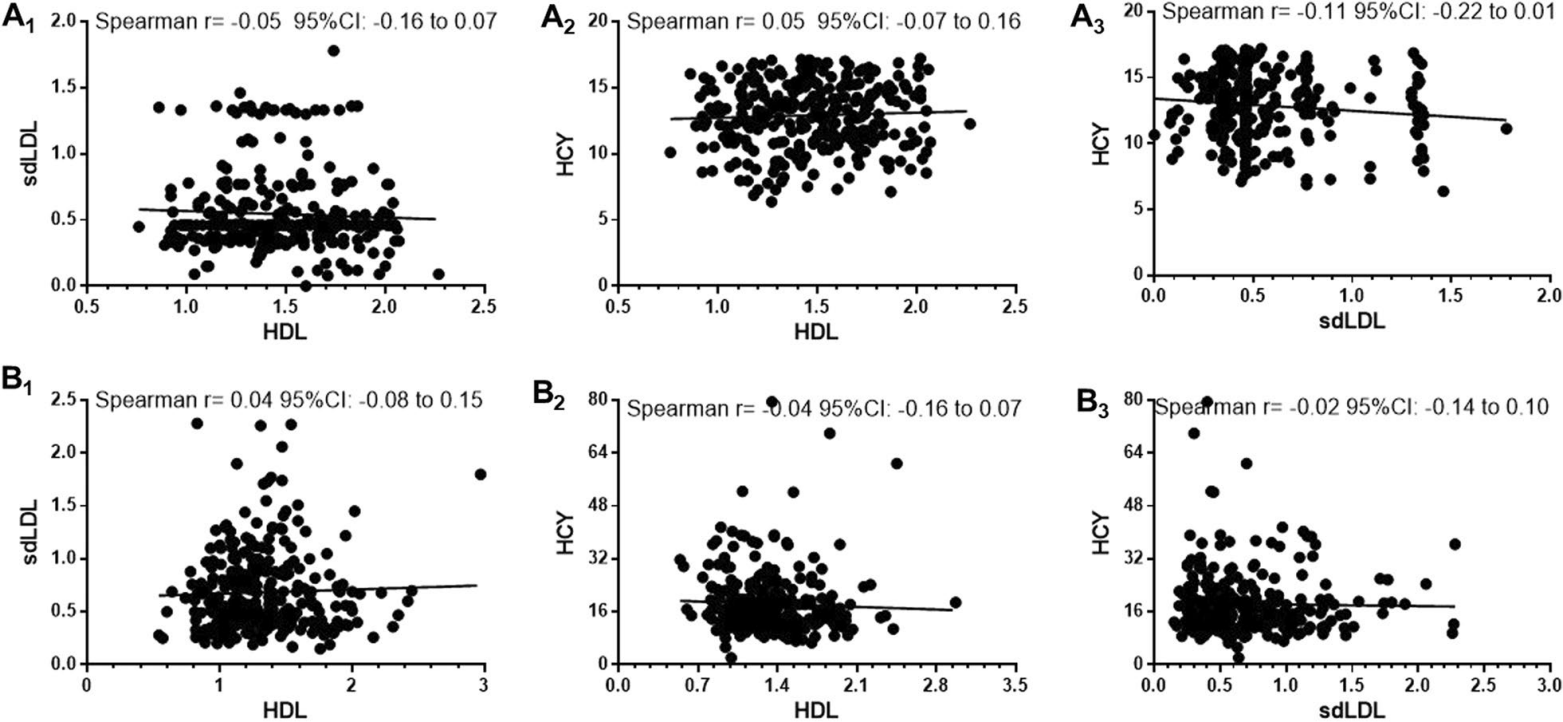
Correlation among three biomarker pairs in two groups. Note: control group (A1, A2 and A3) and cerebral infarction group (B1, B2 and B3). Statistical significance indicated by *p < 0.05

As shown in Fig. 2, spearman analysis revealed that no correlation between plasma sdLDL level and plasma HCY level in control group and cerebral infarction group (r = − 0.11/− 0.02). Plasma HDL level was no correlated with plasma HCY level in control group and cerebral infarction group (r = 0.05/− 0.04). Plasma HDL level was also no correlated with plasma sdLDL in control group and cerebral infarction group (r = − 0.05/0.04). Therefore, the three markers are independent of each other.

### Prediction ability of stroke risk based on three biomarkers

**Fig. 3:**
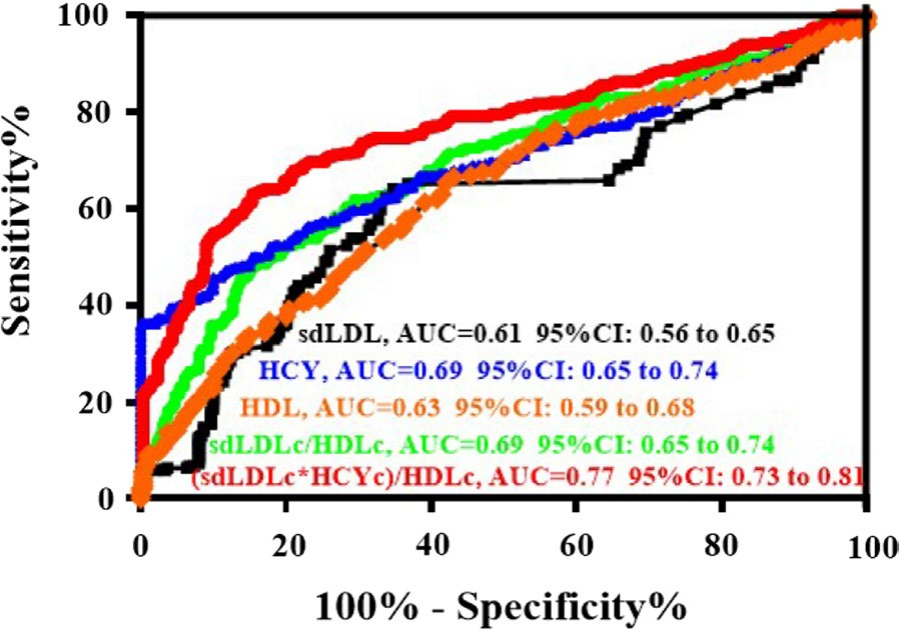
ROC curve analysis of the predictive value of sdLDL, HCY, HDL, sdLDLc/HDLc and (sdLDLc*HCYc)/HDLc

AS shown in Table 2, the positive predictive value and negative predictive value for cerebral infarction based on single biomarker (sdLDL, HCY or HDL) are 6.69%, (95% CI: 4.19–10.42) and 98.66% (95% CI: 96.38–99.57), 38.38% (95% CI: 32.75–44.33) and 97.66% (95% CI: 95.03–98.97), 3.87% (95% CI: 2.05–7.82) and 100% (95% CI: 98.43–100), respectively. The positive predictive value and negative predictive value for cerebral infarction based on two markers (both sdLDL and HDL) in combination by calculating sdLDLc/HDLc is 49.56%, 95% CI: 45.40–53.73 and 84.43%, 95% CI: 79.61–88.31. The positive predictive value and negative predictive value for cerebral infarction based on three biomarkers (sdLDL, HCY and HDL) in combination by calculating (sdLDLc*HCYc)/HDLc is 61.27%, 95% CI: 55.31–66.92 and 84.95%, 95% CI: 80.27–88.74. Risk prediction receiver operating characteristic (ROC) curve was also established (Fig. 3). With an AUC of 0.77 (95% CI 0.73–0.81), (sdLDLc*HCYc)/HDLc showed a significantly greater discriminatory ability, compared with sdLDL (AUC 0.61; 95% CI: 0.56–0.65), HCY (AUC 0.69; 95% CI: 0.65–0.74), HDL (AUC 0.63; 95% CI: 0.59–0.68) and sdLDL/HDL (AUC 0.69; 95% CI: 0.65–0.74), in primary cerebral infarction detection.

**Table 2:** Comparison discriminatory diagnosis ability of first cerebral infarction occurrence based on single biomarker and (sdLDLc*HCYc)/HDLc ratio

	Positive predictive value	95% CI	Negative predictive value	95% CI
sdLDL	6.69	4.19–10.42	98.66	96.38–99.57
HCY	38.38	32.75–44.33	97.66	95.03–98.97
HDL	3.87	2.05–7.02	100	98.43–100
sdLDLc/HDLc	49.56	45.40–53.73	84.43	79.61–88.31
(sdLDLc*HCYc)/HDLc	61.27	55.31–66.92	84.95	80.27–88.74

## Discussion

Over the last decade, China has witnessed a dramatic increase in the incidence of cerebral infarction with high morbidity and mortality rates, which might be caused by changes on lifestyle behavior. It is still hard to take effective way to reduce the mortality of acute cerebral infarction. However, many patients diagnosed as primary cerebral infarction have developed into acute type. Therefore, timely diagnosis of cerebral infarction is of vital importance in planning intervention effect of rapid rehabilitation. Traditional risk factors are not always enough to predict or diagnose the incidence of cerebral infarction. In the present study, results (shown in Table 2 and Fig. 3) illustrate that use of three biomarkers in combination by calculating (sdLDLc*HCYc)/HDLc ratio could improve the diagnosis ability for primary cerebral infarction.

Our current results (Table 1) show that middle-old aged male is likely to develop cerebral infarction. This indicates that gender still has an impact on the risk of cerebral infarction. Many reports have pointed out that men are affected by smoking, drinking and obesity, and these factors could illustrate the phenomenon that cerebral infarction increases with male [16, 17].

sdLDL is considered risk factor for cardiovascular disease owing to its ability to aggregate large amounts of cholesterol in the artery intima. Therefore, sdLDLc could serve as a key risk factor in predicting the incidence of cardiovascular disease. Some reports have illustrated that sdLDLc was higher in the cerebral infarction group than that in non-cerebral infarction group (*p* < 0.05) [9, 18]. Here, our findings that sdLDL levels were significantly higher in the experiment group than in the control group, which is consistent with recent reports. Through analysis, we found that sdLDLs have certain predictive value for diagnosis of primary cerebral infarction.

In contrast, HDL could resist the accumulation, preservation and oxidation of LDL, and serve as a protective factor. The HDLc could affect the development of acute ischemic stroke and a low HDL cholesterol concentration could be found in cerebral infarction population (shown in Table 1) [19]. HDL combined with other biomarkers has a good predictive value for stroke cardiovascular disease. As shown in Table 1, platelets, lymphocytes, neutrophils and monocytes are significantly different between patients in cerebral infarction group and healthy people in control group. It is reported that monocyte/high-density lipoprotein has a good predictive value for cardiovascular disease [20]. Our results show that the monocyte/high-density lipoprotein ratio in cerebral infarction group is significantly higher than that in control group (Data not shown). Previous studies also have suggested that LDLc to HDLc ratio (LDLc/HDLc) is significantly associated with acute coronary syndromes [12]. In current study, higher sdLDLc/HDLc ratio was still significantly associated with increased presence of carotid plaques, indicating that the sdLDLc/HDLc ratio might deserve more attention for these high-stoke-risk population. Our results also indicated that sdLDLc/HDLc ratio could be a better prediction indicator than single biomarker, such sdLDL, HDL (shown in Table 2).

In the 1980s, it was proposed that HCY is an independent risk factor for atherosclerosis and coronary heart disease [21]. HCY was also an independent risk factor of cerebral infarction. Our study shows that compared with participants with health people, serum HCY level in first cerebral infarction continues to increase, which indicates that HCY is the risk factor of cerebral infarction and might be as the biomarker of first cerebral infarction judgement. These conclusions have been confirmed by current studies [22, 23].

Analysis based on ROC curve shows that (sdLDLc*HCYc)/HDLc ratio has better predictive value for first cerebral infarction. (sdLDLc*HCYc)/HDLc ratio might be better indicator in clinical auxiliary diagnosis of primary cerebral infarction than single biomarker, including sdLDLc, HCYc and HDLc, even two biomarkers (sdLDLc/HDLc ratio). This better diagnostic ability may result from taking homocysteine into account. For people with different (sdLDLc*HCYc)/HDLc ratio, we should pay more attention to the changes of thrombus and other indicators, which might help clinician to provide them with thrombolytic, oral anticoagulant and other different treatments based on above findings.

Nevertheless, the present work has some limitations. Firstly, the volume of specimens included was not large. Then, the dynamic monitoring of (sdLDLc*HCYc)/HDLc variation might be more helpful in judging the condition and evaluating the prognosis of cerebral infarction. In addition, the paper shows that there are obvious differences in age and hemogram between the population in control group and the population in first cerebral infarction. These biomarkers, including platelets, lymphocytes, neutrophils and monocytes, were good predictors for cerebrovascular events [24]. In the future, more large-scale research is needed to establish a model based on these indicators to predict cerebral infarction.

In conclusion, three biomarkers in combination by calculating (sdLDLc*HCYc)/HDLc ratio is closely associated with the occurrence of cerebral infarction and could improve the diagnosis ability of single biomarker, including sdLDLc, HCYc and HDLc, for primary cerebral infarction. The new indicator could predict the occurrence of cerebral infarction and provide a scientific basis for risk stratification management and early prevention in people with high risk of stroke due to cerebral infarction.

## Data Availability

The datasets used and/or analysed during the current study are available from the corresponding author on reasonable request.

## Abbreviations

sdLDL: Small dense low-density lipoprotein
HCY: Homocysteine
HDL: High-density lipoprotein cholesterol
LDL: Low-density lipoprotein
TG: Triglyceride
TC: Total cholesterol
GLU: Glucose
UA: Uric acid
hsCRP: Hypersensitive-c-reactive-protein
HbA1C: Glycosylated Hemoglobin
LP-PLA2: Lipoprotein-associated phospholipase A2
CVDs: Cardiovascular disease
CAD: Coronary artery disease
sdLDLc: Small dense low-density lipoprotein concentration
HCYc: Homocysteine concentration
HDLc: High-density lipoprotein cholesterol concentration
ROC: Receiver-operating characteristic curve
